# Supporting data and methods for the characterization of iron oxide nanoparticles conjugated with pH-(low)-insertion peptide, testing their cytotoxicity and analyses of biodistribution in SCID mice bearing MDA-MB231 tumor^☆^

**DOI:** 10.1016/j.dib.2019.105062

**Published:** 2019-12-31

**Authors:** Alexandra G. Pershina, Olga Ya Brikunova, Natalya A. Perekucha, Alexander M. Demin, Oleg B. Shevelev, Dina Malkeyeva, Elena Kiseleva, Artem S. Minin, Larisa A. Kostikova, Ivan V. Stepanov, Dmitriy K. Kuznetsov, Vladimir Ya Shur, Victor P. Krasnov

**Affiliations:** a Siberian State Medical University, 2, Moskovsky trakt, 634050, Tomsk, Russia; b Research School of Chemistry and Applied Biomedical Sciences, National Research Tomsk Polytechnic University, 30, Lenin Ave., Tomsk, 634050, Russia; c Postovsky Institute of Organic Synthesis UB RAS, 22, S. Kovalevskaya St., 620990, Yekaterinburg, Russia; d Institute of Cytology and Genetics SB RAS, 10, Lavrentiev Ave., 630090, Novosibirsk, Russia; e Miheev Institute of Metal Physics UB RAS, 18, S. Kovalevskaya St., 620990, Yekaterinburg, Russia; f Institute of Natural Sciences and Mathematics, Ural Federal University, 51, Lenin Ave., 620000, Yekaterinburg, Russia

**Keywords:** pHLIP, Iron oxide magnetic nanoparticle, Nanoparticle characterization, Tumor, Biodistribution, Cytotoxicity

## Abstract

The method of Fe_3_O_4_ magnetic nanoparticle synthesis by co-precipitation, modification by 3-aminopropylsilane and conjugation with pH-(low)-insertion peptide (pHLIP) is reported. The characterization of nanoparticles by scanning electron microscopy, transmission electron microscopy, Fourier-transform infrared spectroscopy, elemental and thermogravimetric analyses as well as dynamic light scattering and z-potential measurements is provided. The effect of nanoparticles on the viability of mouse and human peripheral blood mononuclear cells is tested by flow cytometry. The experimental details of nanoparticle administration to tumor-bearing mice, magnetic resonance imaging scanning as well as subsequent tumor sample collection and their processing for transmission electron microscopy, inductively coupled plasma atomic emission spectroscopy, histological and immunohistochemical analyses are described. Biodistribution of the nanoparticles in mice and blood serum analysis data for experimental animals are given. The data are useful for an experiment workflow design and for the development of theranostic systems based on magnetic nanoparticles.

**Table:** Specifications Table

Subject	Biology
Specific subject area	Chemistry, biology and material science
Type of data	Table, scheme, image, graph, figure
How data were acquired	The morphology and size of nanoparticles were characterized by transmission electron microscopy (TEM) and scanning electron microscopy (SEM). The hydrodynamic size of the nanoparticles was measured by dynamic light scattering (DLS). The immobilization of pHLIP on magnetic nanoparticles was confirmed by FTIR spectroscopy. The number of organic molecules bound to the nanoparticle surface was calculated based on Fourier-transform infrared (FTIR) spectroscopy, elemental analysis (CHN), inductively coupled plasma atomic emission spectroscopy (ICP-AES) and thermogravimetric analysis (TGA) data. Cell viability was measured by flow cytometry using membrane-impermeant DNA-binding fluorescent dyes. The concentration of iron in the tissue was measured by ICP-AES. The amount of eosinophils in tumor microsection was quantified by TEM. Perls' Prussian blue and hematoxylin & eosin stained tissue sections were imaged by light microscopy. Serum biochemical parameters were determined using clinical chemistry analyzer. Instruments: Nicolet 6700 Thermo FTIR-spectrometer (Thermo Scientific), CHN РЕ 2400 II automatic analyzer (Perkin Elmer), TGA/DSC1 instrument (Mettler Toledo), Zetasizer Nano ZS (Malvern), Merlin scanning electron microscope (SEM) (Carl Zeiss) equipped with Gemini column with Schottky field emission cathode as an electron source, ARCHITECT c4000 (Abbott Diagnostics, Lake Forest), BD Accuri-C6 flow cytometer (BD Bioscience), Horizontal tomographic scanner Biospec 117/16 USR (Bruker), iCAP 6300 Duo ICP Spectrometer (Thermo Scientific), Excelsior™ AS Tissue Processor (Thermo Scientific), HM 325 Rotary Microtome (Thermo Scientific), BOND-MAX Automated IHC/ISH Stainer (Leica Biosystems), Leica ultracut ultra-microtome (Leica), Transmission electron microscope JEM-1400 (JEOL). Data were analyzed using AxioVision, GraphPad Prism GraphPad Software.
Data format	Raw and analyzed.
Parameters for data collection	MDA-MB231 xenografts were established in 4–6 week old female SCID (SHO-PrkdcscidHrhr) mice. MNP-APS and MNP-pHLIP were administered by the retro-orbital route. PBMCs were isolated by density gradient centrifugation from the blood of healthy donors or from heparinized mouse blood obtained by cardiac puncture.
Description of data collection	Nanoparticle samples for SEM, FTIR, TGA, elemental analysis were evaporated from their colloidal solutions. High-resolution T2-weighted MR images of tumors in mice were acquired with respiratory triggering using TurboRARE T2. Blood samples were collected in Microvette® CB 300. The liver, spleen, kidney, lung, thymus, lymph nodes, and tumor were dissected, cut into pieces and frozen at −80 °C, placed in 10% neutral buffered formalin or cold 2.5% glutaraldehyde.
Data source location	Siberian State Medical University, Tomsk, Russia.
Data accessibility	The raw data are provided as supplementary file and are available from the corresponding author upon reasonable request.
Related research article	Alexandra G. Pershina, Olga Ya. Brikunova, Alexander M. Demin, Oleg B. Shevelev, Ivan A. Razumov, Evgenii L. Zavjalov, Dina Malkeyeva, Elena Kiseleva, Nadezhda V. Krakhmal’, Sergey V. Vtorushin, Vasily L. Yarnykh, Vladimir V. Ivanov, Raisa I. Pleshko, Victor P. Krasnov, Ludmila M. Ogorodova pH-Triggered Delivery of Magnetic Nanoparticles Depends on Tumor Volume Nanomedicine: Nanotechnology, Biology and Medicine DOI: 10.1016/j.nano.2019.102086 [1]

**Table:** 

**Value of the Data** • A comprehensive characterization of nanomaterial based on magnetic nanoparticles conjugated with pH-low-insertion peptide is provided. • The protocols used for *in vitro* and *in vivo* experiments are extensively described. • These data are valuable for the design of nanomaterials for theranostics.

## 1 Data

**Fig. 1:**
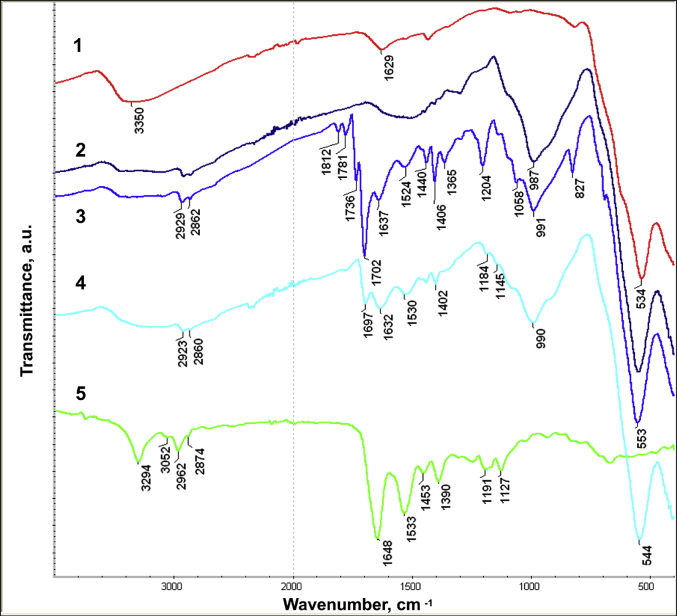
FTIR spectra of initial magnetic nanoparticles (MNP) (1), MNP-APS (2), MNP-EMCS (3), MNP-pHLIP (4), and pHLIP (5). The characteristic absorption bands of MNP-APS (544 cm^−1^, Fe–O; 990 cm^−1^, Si–O) and the slightly shifted bands attributed to the peptide (1632 cm^−1^; C=O (amide I), 1530 cm^−1^ C–N (amide II)), and the band at 1697 cm^−1^ corresponding to the stretching vibrations of C=O of the cross-linker are presented on MNP-pHLIP FTIR spectrum.

**Fig. 2:**
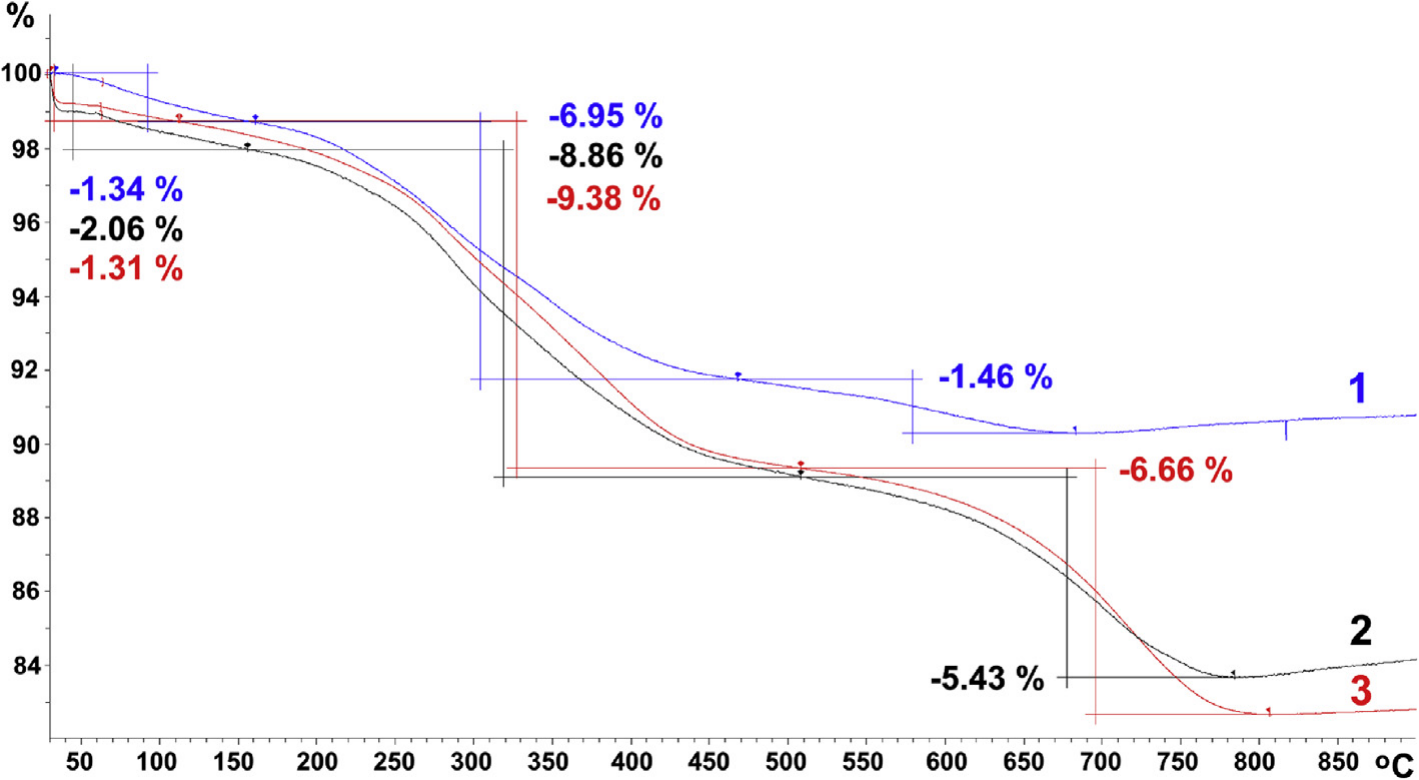
TGA data of MNP-APS (1), MNP-EMCS (2) and MNP-pHLIP (3). The total weight loss for samples 1–3, due to decomposition of the organic shell, was 8.41, 14.29, 16.04 mg/g MNPs, respectively.

**Fig. 3:**
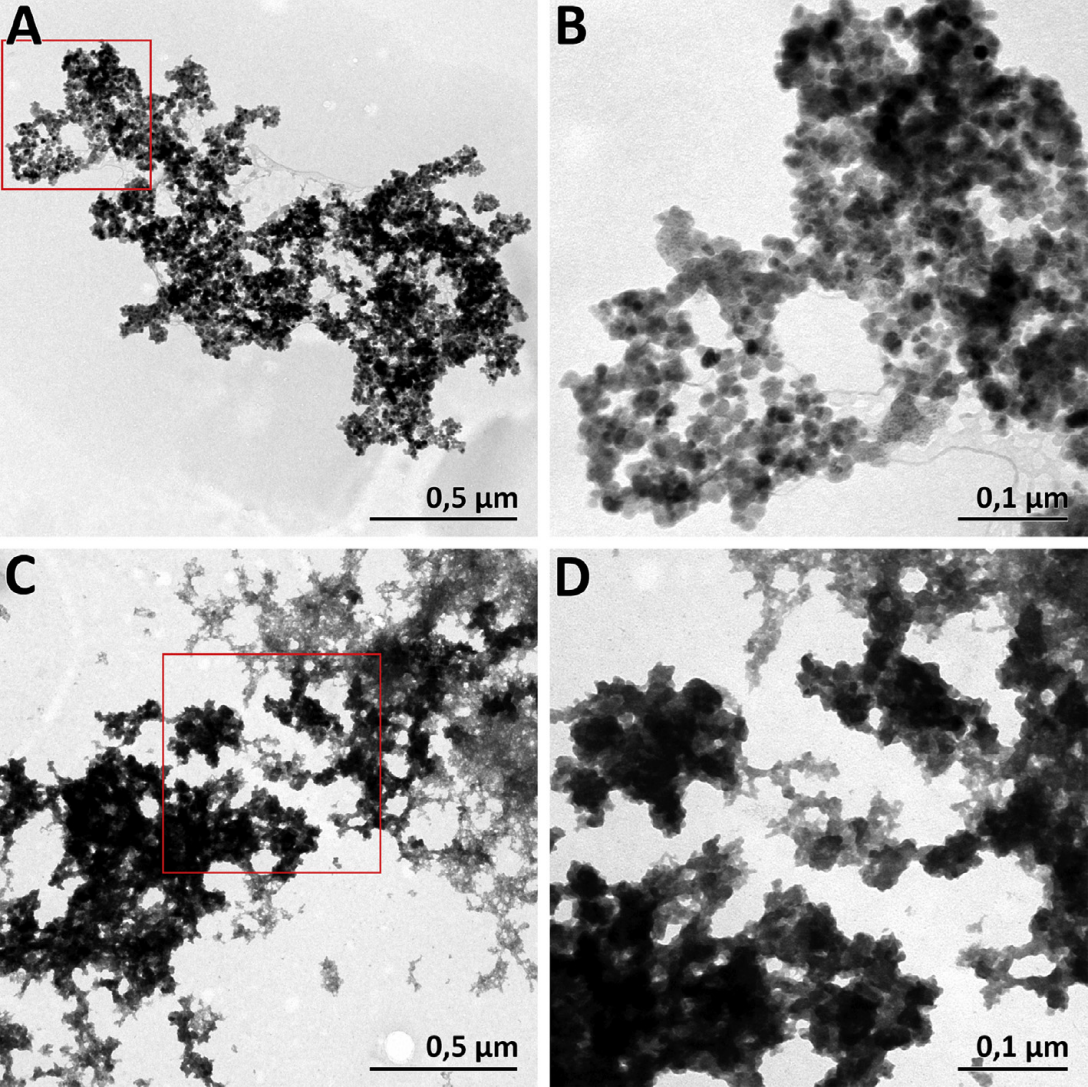
TEM images of (A, B) MNP-APS and (C, D) MNP-pHLIP. (B) Region magnified from red square on A. (D) Region magnified from red square on C. The parent MNP-APS particles were spherical or truncated polygonal in shape, varying from approximately 7 to 18 nm in size. The MNP-pHLIP represent nanoparticles surrounded by amorphous matrix.

**Fig. 4:**
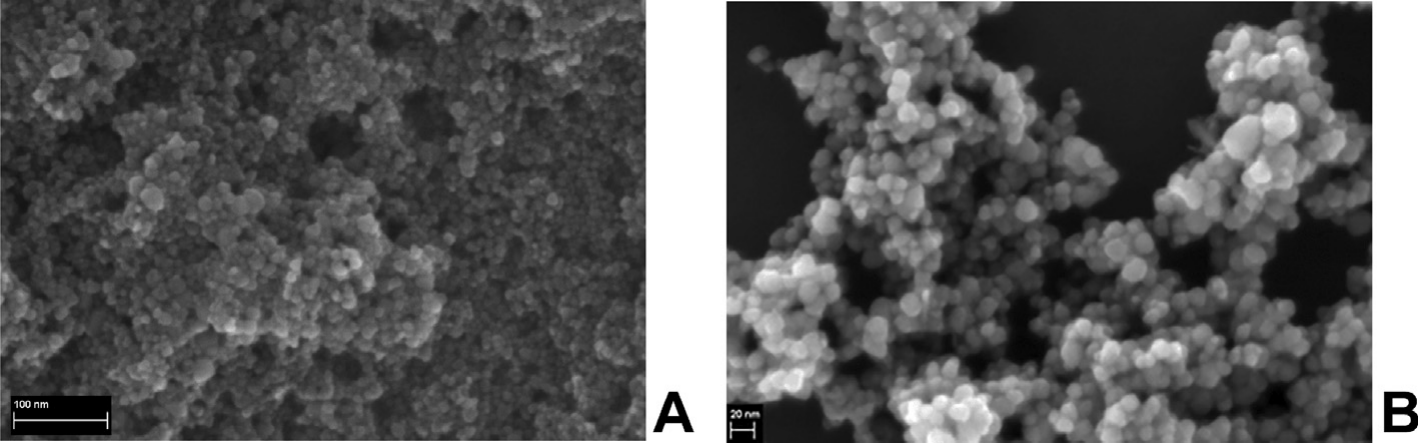
SEM images of (A) MNP-APS and (B) MNP-pHLIP.

**Fig. 5:**
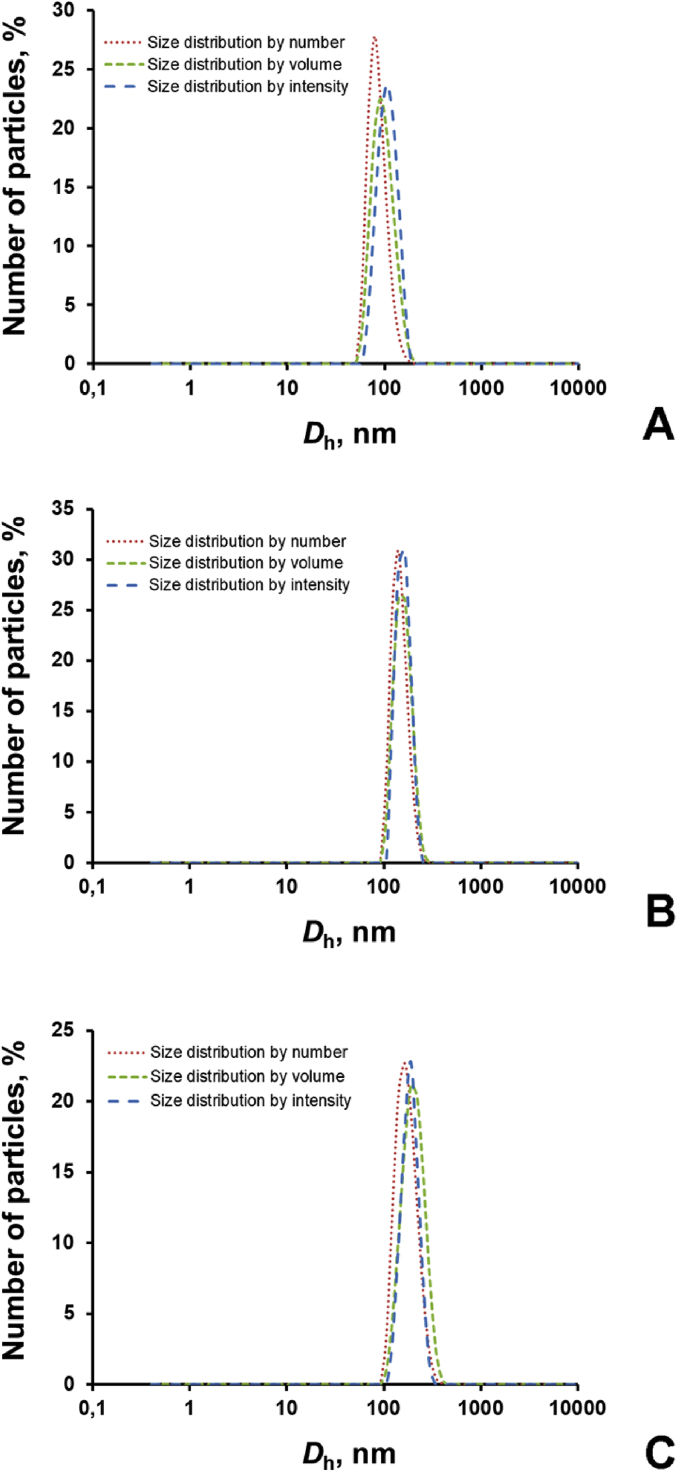
Dynamic light scattering (DLS) size distributions (*D*_h_) for (A) MNP-APS in water and (B) MNP-pHLIP in water and (C) MNP-pHLIP in DMEM supplemented with 10% fetal bovine serum (FBS).

**Fig. 6:**
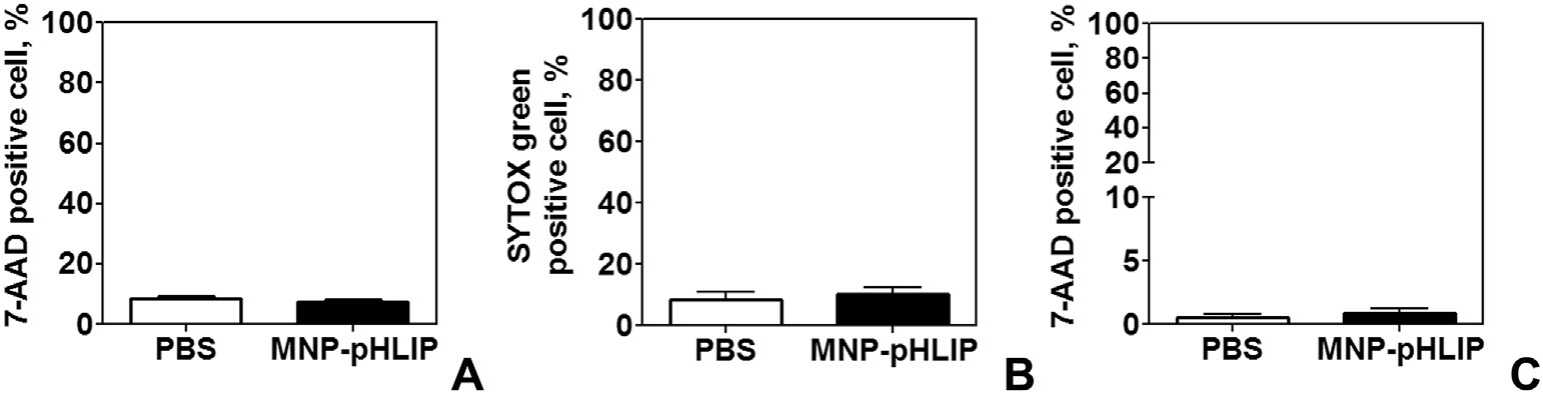
Effect of MNP-pHLIP on blood cells viability. The amount of (A) necrotic mouse peripheral blood mononuclear cells (PBMCs) after incubation for 2 hours with MNP-pHLIP (20 μg [Fe]/mL), (B) necrotic human and (C) mouse monocytes after incubation for 24 hours with MNP-pHLIP (20 μg [Fe]/mL) according to 7-aminoactinomycin D (7-AAD) or SYTOX Green flow cytometry analysis. Data are shown as the mean ± SD.

**Fig. 7:**
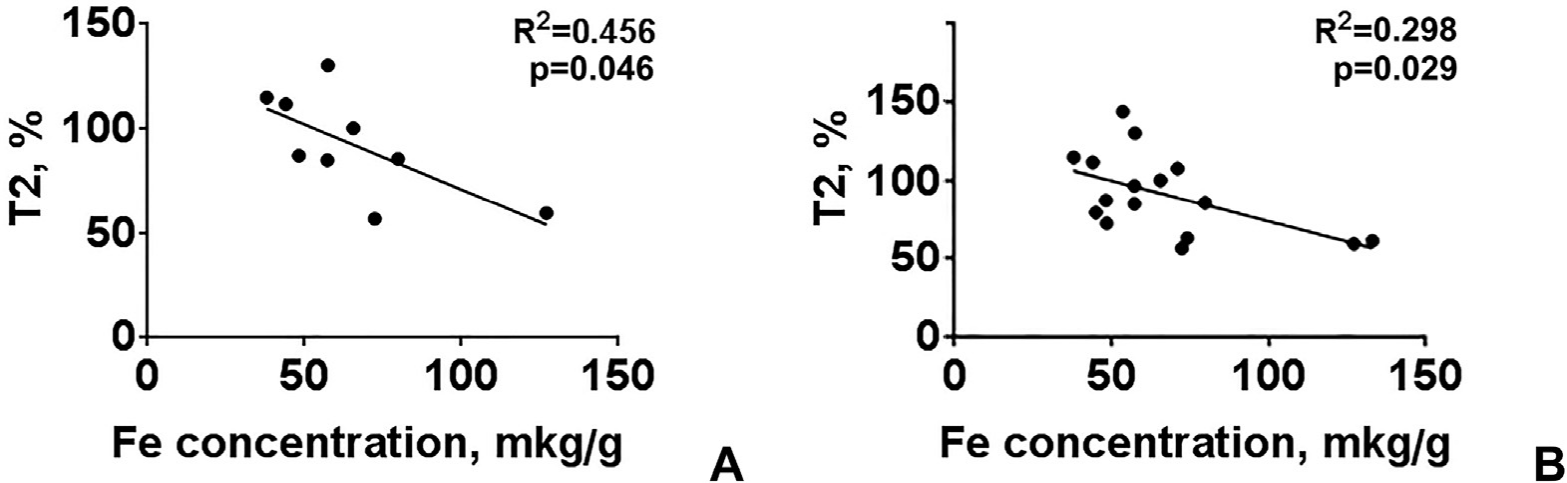
The dependence of SI changes in T2-weighed MR images on iron concentration in the tumors (A) in group of mice administered with MNP-pHLIP, (B) in two groups of mice administered with MNP-APS and MNP-pHLIP.

**Fig. 8:**
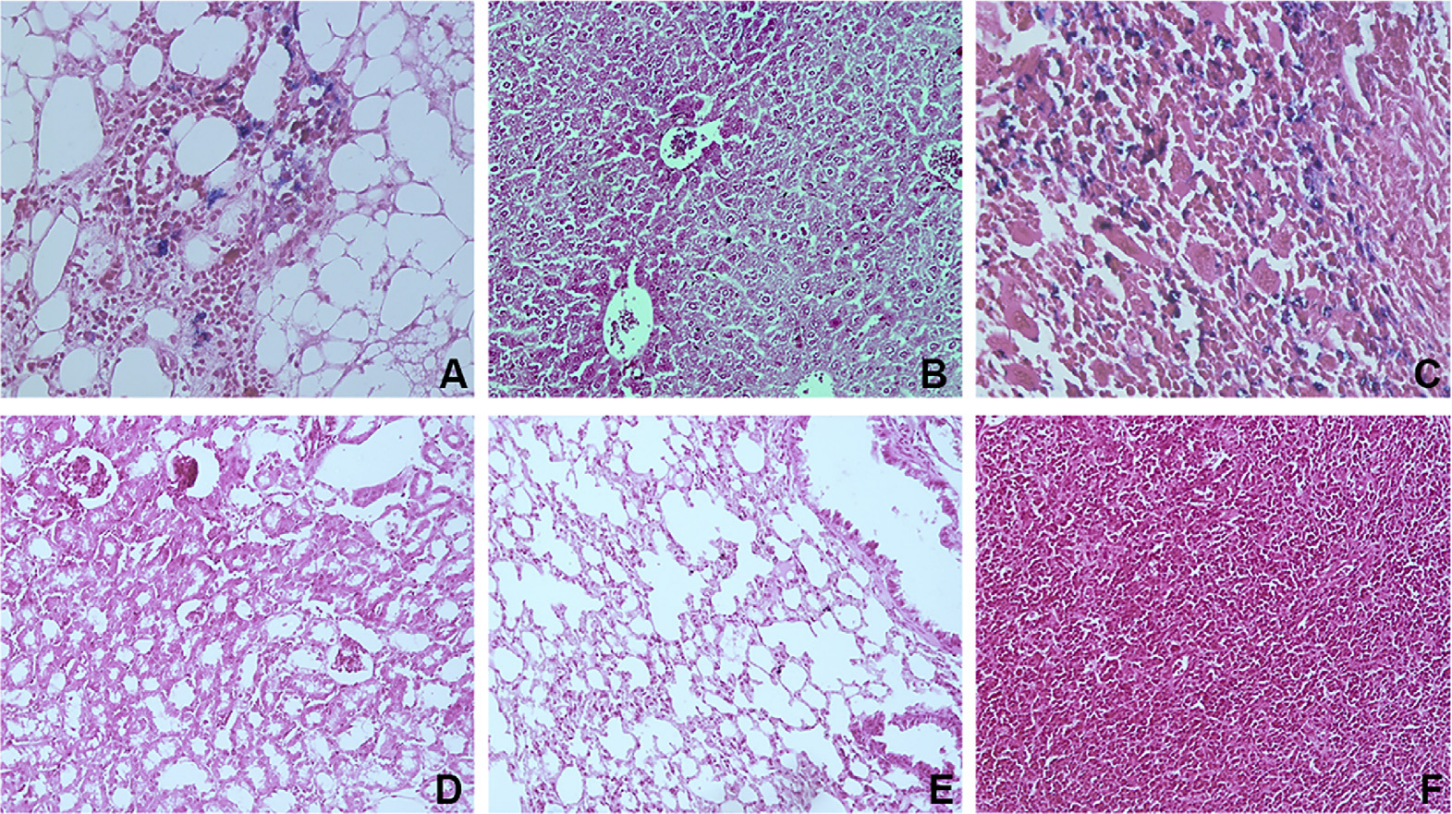
Histological examination of (A) right inguinal lymph node with perinodal adipose tissue, (B) liver, (C) spleen, (D) kidney (E) lung and (F) intact lymph node of MDA-MB231 tumor-bearing mice extracted 40 h after MNP-pHLIP administration. Perls' positive cells was identified in metastasis in the regional lymph node isolated from one mouse injected with MNP-pHLIP. Notably, hardly any Perls' positive cells were found in the liver. Perls' positive cells are identified in splenic macrophages. Perls' Prussian blue staining, shown at a × 400 magnification.

**Fig. 9:**
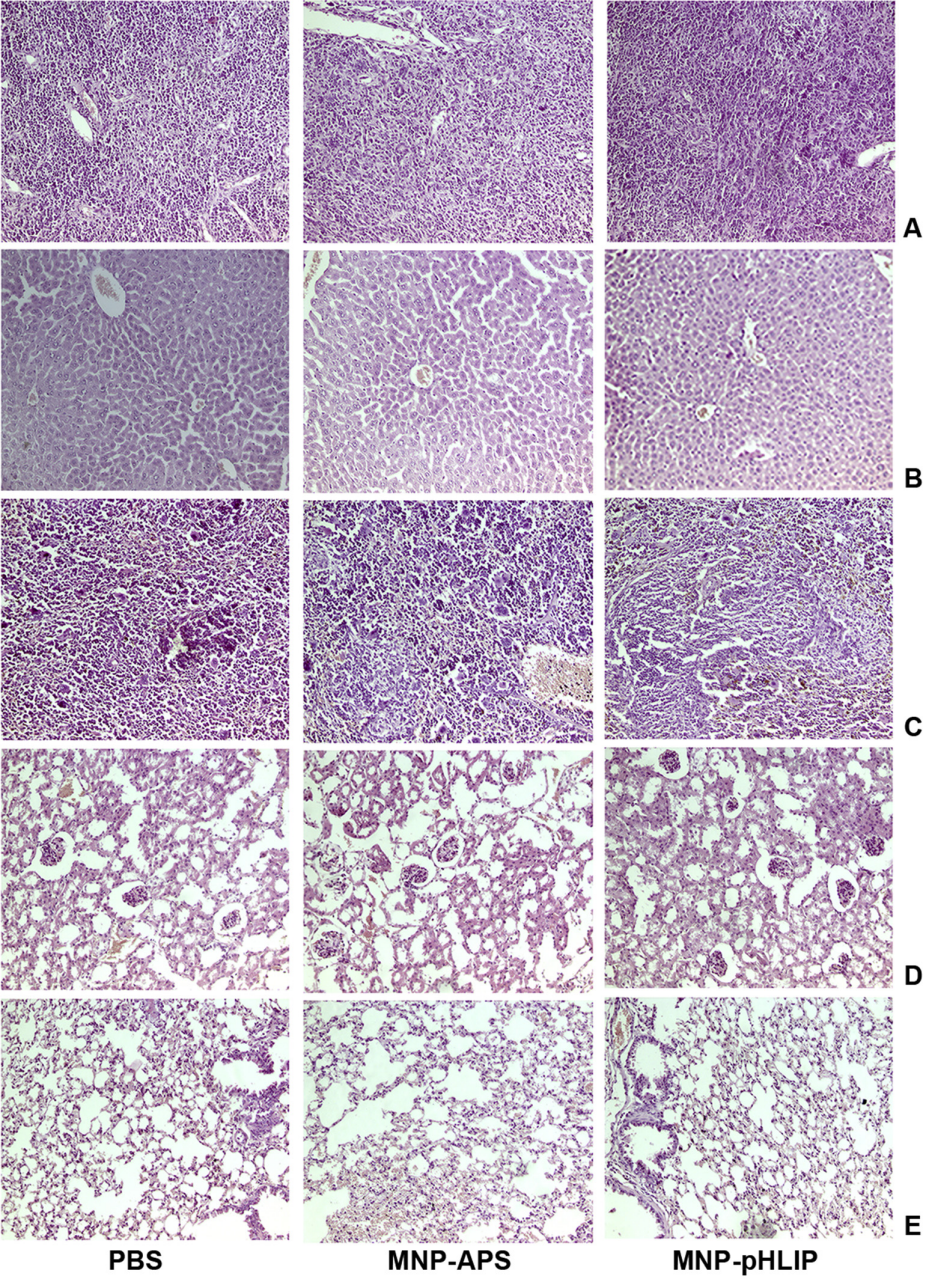
Histological examination of (A) the thymus (B) liver, (C) spleen, (D) kidney, (E) lung of MDA-MB231 tumor-bearing mice extracted 40 h after PBS, MNP-APS or MNP-pHLIP administration. Hematoxylin & eosin staining, shown at a × 400 magnification.

The dataset in this article describes the method of Fe_3_O_4_ magnetic nanoparticle (MNP) synthesis by co-precipitation, its modification by 3-aminopropylsilane (APS) and conjugation with pH-(low)-insertion peptide (pHLIP), the experimental details of the investigation of MNP-pHLIP interaction with cells *in vitro*, the experimental details of nanoparticles administration to tumor-bearing mice, magnetic resonance imaging (MRI) scanning, tumor sample collection and their processing for TEM and for inductively coupled plasma atomic emission spectroscopy, histological and immunohistochemical analyses. Fig. 1 describes the FTIR spectra of modified nanoparticles. Table 1 describes the elemental analysis data for the modified nanoparticles. Fig. 2 describes the TGA data for the modified nanoparticles. Fig. 3 provides the transmission electron microscopy (TEM) images of the modified nanoparticles. Fig. 4 provides the scanning electron microscopy (SEM) images of MNP-APS and MNP-pHLIP. Fig. 5 and Table 2 describe the data of dynamic light scattering (DLS) analysis for colloidal solutions of MNP-APS and MNP-pHLIP. Fig. 6 describes the effects of MNP-pHLIP on blood cell viability *in vitro*. Fig. 7 describes the dependence of signal intensity change in T2-weighted MR image on iron concentration in the tumor 40 hours after nanoparticles administration to mice. Fig. 8 provides Perls' Prussian blue stained sections of liver, spleen, kidney, lung, right inguinal and intact lymph nodes of MDA-MB231 tumor-bearing mice administered with MNP-pHLIP. Fig. 9 provides hematoxylin and eosin stained sections of thymus, liver, spleen, kidney and lung of experimental animals. Table 3 describes the quantification of eosinophils in tumor microsection by TEM. Table 4 provides the data for iron concentration in the samples of tissue extracted from experimental mice according to ICP-AES. Table 5 provides serum biochemical parameters of the experimental mice.

**Table 1:** The elemental analysis data for MNP-APS, MNP-EMCS and MNP-pHLIP.

	MNP-APS	MNP-EMCS	MNP-pHLIP
C, %	2.69	4.69	6.23
*c*, mmol/g MNPs	0.71/0.72^a^/0.68^b^	0.15	0.0035

a The amount of APS immobilized on the MNP surface calculated according to FTIR data.

b The amount of APS immobilized on the MNP surface calculated according to ICP-AES data.

**Table 2:** DLS analysis of MNP-APS and MNP-pHLIP.

	Medium	Types of size distribution	*D*_h_, nm	z*P*, mV	PdI	Characters of size distribution
MNP-APS	DI water	by number	79	+29.1	0.16	monomodal
		by volume	91			monomodal
		by intensity	109			monomodal
MNP-pHLIP	DI water	by number	142	−22.1	0.10	monomodal
		by volume	142			monomodal
		by intensity	164			monomodal
MNP-pHLIP	DMEM,10% FBS	by number	142	−9.6	0.19	monomodal
		by volume	142			monomodal
		by intensity	190			monomodal

**Table 3:** Quantification of eosinophils in tumor microsection by TEM.

	Control			MNP-APS			MNP-pHLIP			
# of sample	1	2	3	1	2	3	1	2	3	4
N (cell)	50	50	50	50	50	50	50	50	50	50
N (eosinophil)	0	0	0	2	5	1	5	1	1	0
Eosinophils, %	**0.00**	**0.000**	**0.00**	**4.00**	**10.00**	**2.00**	**10.00**	**2.00**	**2.00**	**0.00**

**Table 4:** Concentration of Fe (mg/g) in the samples of tissue extracted from PBS, MNP-APS and MNP-pHLIP administered mice according to ICP-AES data.

Tissue type	Control n = 9		MNP-APS n = 9		MNP-pHLIP n = 9	
	mean	SD	mean	SD	mean	SD
Kidney	0.13	0.02	0.13	0.04	0.15	0.05
Spleen	0.98	0.40	1.14	0.31	1.28	0.38
Liver	0.26	0.03	0.23	0.04	0.23	0.03
Lung	0.22	0.04	0.21	0.07	0.20	0.05
Thymus	0.18	0.08	0.21	0.11	0.16	0.07

**Table 5:** Serum analysis of the MDA-MB231 tumor-bearing mice 40 h after PBS (control), MNP-APS and MNP-pHLIP administration.

Parameter	Control n = 9		MNP-APS n = 9		MNP-pHLIP n = 9	
	mean	SE	mean	SE	mean	SE
ALT, U/L	74.78	16.33	75.67	13.14	66.00	12.19
AST, U/L	119.00	10.81	139.20	12.57	146.10	11.92
Urea, U/L	8.08	0.39	8.32	0.37	9.00	0.48
Creatinine, umol/L	30.35	0.83	29.38	0.49	29.51	0.55
Albumin, g/L	31.25	0.31	31.56	0.47	32.33	0.78

AbbreviationsALT, Alanine transaminase; AST, Aspartate aminotransferase; ALP, Alkaline phosphatase.

## 2 Experimental design, materials, and methods

### 2.1 Synthesis of nanomaterials

**Scheme 1:**
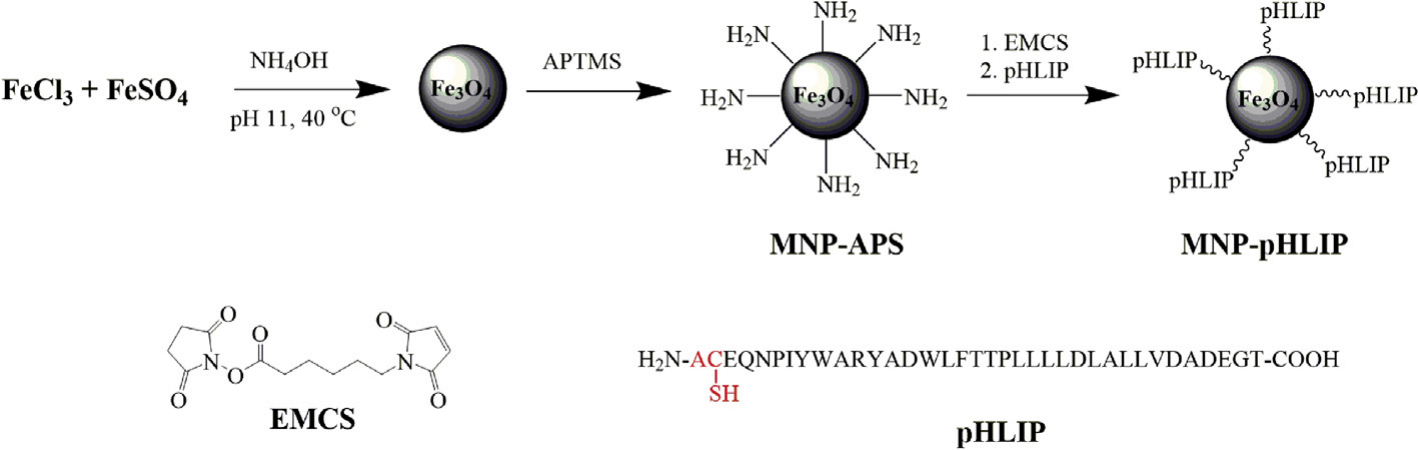
Immobilization of pHLIP on MNP surface.

Initial Fe_3_O_4_ magnetic nanoparticles were obtained by co-precipitation method [2,3]. The modification of Fe_3_O_4_ nanoparticles with 3-aminopropylsilane was carried out according to Ref. [2] and with pHLIP peptide according to Refs. [4,5] (Scheme 1). (3-Aminopropyl) trimethoxysilane (APTMS, 97%; Alfa Aesar, England), 6-maleimidohexanoic acid *N*-hydroxysuccinimide ester (EMCS; Fluka, USA), pH-low insertion peptide (NH_2_-ACEQNPIYWARYADWLFTTPLLLLDLALLVDADEGT-COOH) (pHLIP; Bachem, USA) were applied. All other chemicals of analytical grade were purchased from commercial suppliers and used as received. Deionized (DI) water (18.2 M Ω cm^−1^) and phosphate-buffered saline (PBS, pH 7.4) were used in the experiments.

#### 2.1.1 Preparation of Fe_3_O_4_ MNP

A mixture of FeSO_4_·7H_2_O (0.364 g, 1.31 mmol) and FeCl_3_·6H_2_O (0.714 g, 2.62 mmol) were dissolved in 50 mL of water and heated to 40 °C under argon flow. Then 3 mL of the saturated aqueous ammonia solution was added to the resulting solution up to pH 11 under vigorous stirring. After 10 min the resulting MNPs were precipitated by an external magnet, washed with DI water up to neutral pH and resuspended in EtOH.

#### 2.1.2 Preparation of APS-modified MNPs (MNP-APS)

A solution of APTMS (111.6 μL, 0.635 mmol) in EtOH (5 mL) was added to a solution of MNPs (0.210 g) in 50% EtOH (70 mL) under stirring and heated to 40 °C for 8 h. The resulting MNP-APS were precipitated by an external magnet, washed with EtOH and resuspended in MeCN.

#### 2.1.3 EMCS-functionalization of MNP-APS (MNP-EMCS)

A solution of EMCS (15.8 mg, 0.051 mmol) in MeCN (3 mL) was added to a solution of MNP-APS (70 mg) in MeCN (35 mL), and the resulting mixture was stirred at room temperature for 3.5 h. The resulting MNP-EMCS was centrifuged (18,000 rpm; 10 min), and subsequently washed with MeCN and PBS.

#### Immobilization of pHLIP on MNPs (MNP-pHLIP)

The suspension of pHLIP (3.64 mg, 0.885 μmol) in PBS (3 mL) was added while stirring to a dispersion of MNP-EMCS (50 mg) in PBS (30 mL) under argon flow to prevent disulfide pHLIP-derivative formation. After 16 h, MNP-pHLIP was precipitated by an external magnet and washed three times with PBS. The resulting materials were stored in PBS at 4 °C.

### 2.2 Characterization of MNP-pHLIP

#### 2.2.1 FTIR analysis

IR spectra for nanopowders of synthesized materials were recorded on a Nicolet 6700 Thermo FTIR-spectrometer by the attenuated total reflection (ATR) method on the diamond crystal, in the range of 4000–400 cm^−1^ with 128 scans and at 4 cm^−1^ resolution.

The immobilization of pHLIP on the APS-modified MNP surface through 6-maleimidohexanoic acid *N*-hydroxysuccinimide ester was confirmed by FTIR spectroscopy (Fig. 1). The quantitative analysis of 3-aminopropylsilane content in MNP-APS was carried out in accordance with [6]. The FTIR spectra of the analyzed samples were recorded in five replicates. The amount of APS (*c*) on MNP surface (mmol per g of MNPs) was calculated according to the following formula:

(1) $$c=1.036\times x^{-1.279}$$

where *с* is the amount of APS on the nanoparticle surface, mmol per 1 g of MNPs; *x* is the ratio of integrated intensities of Fe–O (in the range of 450–750 cm^−1^) and Si–O (in the range of 770–1160 cm^−1^) absorption bands in the FTIR spectra.

#### 2.2.2 ICP-AES analysis

The amount of APS in MNP-APS was calculated from the Si mass fraction, which was determined by AES on an iCAP 6300 Duo ICP spectrometer (Thermo Scientific) using the intensity of the most sensitive analytical spectral line at 251.6 nm. (Table 1**).**

#### 2.2.3 Elemental analysis

The amount of pHLIP in the synthesized MNP-pHLIP was calculated from the elemental analysis (CHN) data. The mass fraction of carbon was measured using a CHN РЕ 2400 II automatic analyzer (PerkinElmer) (Table 1). Based on this data, the amount of immobilized APS, EMCS and pHLIP was calculated in accordance with method described in Refs. [4,7].

Thermogravimetric analysis. Thermogravimetric analysis (TGA) was carried out on a TGA/DSC1 instrument (Mettler Toledo) with a heating rate of 10 °C min^−1^ and a temperature range of 30–900 °C under compressed Ar (Fig. 2).

#### 2.2.4 TEM

For the analysis of MNP-APS and MNP-pHLIP a drop of nanoparticle suspension was placed onto formvar-coated copper grids. The excess liquid was then removed by filter paper and the sample was left to dry under ambient air, before being analyzed under the transmission electron microscope JEM-1400 (JEOL, Tokyo, Japan) (Fig. 3). In the case of MNP-pHLIP nanoparticles, the samples were preliminarily stained with uranyl acetate and lead citrate.

#### 2.2.5 SEM

Nanoparticles were characterized using Merlin scanning electron microscope (Carl Zeiss, Germany) equipped with Gemini column with Schottky field emission cathode as an electron source. The average diameter of MNP-APS and MNP-pHLIP were 11 and 13 nm respectively (Fig. 4).

#### 2.2.6 DLS

Dynamic light scattering and zeta potential (z*P*) characterization were carried out using Malvern Zetasizer Nano ZS. Average hydrodynamic diameter (*D*_h_) of MNP-APS and MNP-pHLIP was measured by the three different types of size distributions generated by DLS (number-, volume-, and intensity-weighted distribution) for their colloidal solutions (250 mg/L) in DI water and DMEM with 10% fetal bovine serum (Fig. 5, Table 2).

### 2.3 *In vitro* experiments

#### 2.3.1 Interaction of MNP-pHLIP with MDA-MB231 cells

The MDA-MB231 cells (human adenocarcinoma, ATCC) were grown in 5% CO_2_ at 37 °C in complete DMEM/F-12 (HyClone) medium supplemented with 10% (vol/vol) fetal bovine serum (FBS, Biosera), l-glutamine and gentamicin.

For the cytotoxicity assay MNP-pHLIP in PBS was added to 10^5^ adhered MDA-MB231 cells in 24-well plate (1:10 vol/vol) to a final concentration of 4–40 μg [Fe]/mL and incubated for 2, 24 and 48 h. In control wells, an equal volume of PBS instead of MNP-pHLIP was added. After that, cells were washed twice with PBS, detached by TrypLE without phenol red (Thermo Fisher Scientific), centrifuged for 7 min at 2000 rpm (MiniSpin, Eppendorf), and suspended in 200 μL of PBS. Cell viability was measured using 7-aminoactinomycin D (7-aad, Biolegend, San Diego, CA) according to the manufacturer's protocol on a BD Accuri-C6 flow cytometer. 7-AAD was dissolved in 1 mL of DMSO. For analysis, 2 μL of 7-AAD solution was added to 10^5^ cells in PBS buffer.

The ability of MNP-pHLIP to bind MDA-MB231 in a pH-dependent manner was analyzed at pH 6.8 and 7.4. The pH 6.8 medium was prepared by addition of 1 M HEPES, pH 5.6 (Sigma) up to a concentration of 50 mM. MNP-pHLIP in PBS was added to 10^5^ MDA-MB231 cells seeded in 24-well plate (1:10 vol/vol) to a final concentration of 4–20 μg [Fe]/mL and incubated at pH 6.8 or 7.4. After 1 h, cells were washed twice with PBS (pH 6.8/7.4), detached by TrypLE without phenol red (Thermo Fisher Scientific), centrifuged for 7 min at 2000 rpm, and suspended in 200 μL of PBS (pH 6.8/7.4). 50 μL of cell suspension was used for cell counting on BD Accuri C6.

The 150 μL of cell suspension was evaporated, lysed in 80 μL of HCl overnight, and then 520 μL of DI H_2_O was added. The iron concentration was determined using a slightly modified colorimetric ferrozine-based assay [8]. For ferrozine test preparation 1.9 g ammonium acetate (AppliChem), 16 mg ferrozine iron reagent (Acros organics), 1.8 g l-ascorbic acid (Carl Roth GmbH + Co. KG), 13.5 mg neocuproine (Aldrich) were dissolved in 5 mL of DI water. 600 μL of samples were mixed with 60 μL of the ferrozine test solution, transferred to a 96-well plate, and the optical density was measured at 570 nm on a microplate reader (Sunrise, Tecan). FeCl_3_ (Sigma Aldrich) water solution (0.1–2.0 μg/mL) was used for the calibration curve.

For Perls’ staining, 10^6^ cells were seeded in a 6-well plate and incubated with nanoparticles in a final concentration of 10 μg [Fe]/mL in a complete DMEM/F-12 medium at pH 6.8 and 7.4, respectively. After incubation, cells were washed 3 times with PBS (pH 6.8/7.4) and fixed in 4% paraformaldehyde in PBS buffer for 30 min, washed 3 times with DI water, and stained with 4% K_4_ [Fe(CN)_6_] in 4% HCl. The cell nucleus was counterstained by 0.5% neutral red (Sigma-Aldrich) prepared in a sodium acetate buffer (pH 4.8).

#### 2.3.2 Effect of MNP-pHLIP on mouse PBMCs viability

Mouse peripheral blood mononuclear cells (PBMCs) were isolated from BALB/c mouse blood (heparinized) obtained by cardiac puncture. Blood was diluted with HBSS 1:2, layered over Ficoll density gradient (PanEco), centrifuged at 400*g* for 30 min, washed twice with HBSS (Gibco) at 300*g* for 10 min, and resuspended in RPMI (PanEco). 10^5^ PBMCs were mixed with MNP-pHLIP (to final concentration 20 μg [Fe]/mL) in a polypropylene tube and incubated for 2 h at 37 °C on rotator. After this, cells were washed twice with HBSS via centrifugation and resuspended in PBS buffer. Cell viability was measured using 7-aad (Fig. 6A).

#### 2.3.3 Effect of MNP-pHLIP on viability of human/mouse monocytes

Human PBMCs were obtained from the blood of healthy donors using Ficoll (PanEco) density gradient centrifugation at 500*g* for 30 min, washed twice with HBSS (Gibco) by centrifugation at 165*g* for 20 min and resuspended in RPMI. Monocytes were isolated from human or mouse PBMCs similarly using Percoll (GE Healthcare Bio-Sciences AB) double density gradient centrifugation at 2058*g* for 50 minutes at 20 °C, washed twice with HBSS (Gibco) by centrifugation at 659*g* for 15 min and resuspended in RPMI.

The purified human/mouse monocytes were then seeded in 24-well plates at a concentration of 10^5^ cells per well in RPMI 1640 medium supplemented with 10% (vol/vol) fetal bovine serum (complete RPMI), l-glutamine, 2-mercaptoethanol and penicillin-streptomycin. 24 h after cell incubation at 37 °C in 5% CO_2,_ the medium was replaced with 450 μL of fresh complete RPMI medium. A suspension of the MNP-pHLIP was then added to cells (1:10 vol/vol) to a final concentration equal to 20 μg [Fe]/mL; cells were incubated for 24 hours at 37 °C in 5% CO_2_. In control wells, equal volumes of PBS instead MNP-pHLIP were added. Cell viability was measured using membrane-impermeant DNA-binding fluorescent dyes: 7-aad (for mouse monocytes) and SYTOX green (Thermo Fisher Scientific) (for human monocytes) (Fig. 6B and C).

### 2.4 Animal experiments

The animal experiments were conducted in compliance with the Guide for the Care and Use of Laboratory Animal Resources (1996) and approved by the Bioethics Review Committee of the Institute of Cytology and Genetics SB RAS (No. 38 from July 18, 2017).

To establish xenografts, 4–6 week old female SCID (SHO-PrkdcscidHrhr) mice (n = 27) were injected in the right fat pad with 5*10^6^ MDA-MB231 cells suspended in 100 μL of serum free media and matrigel at a 1:1 ratio. The mice were housed in individually ventilated OptiMICE cages (Animal Care Systems) in groups of three animals per cage with ad libitum food (Ssniff) and water. The cages containing the mice were held in a barrier room of SPF animal facility with a regular 14/10 h light/dark cycle (lights on 02:00AM), a constant room temperature of 24 °C ± 2 °C, and a relative humidity of approximately 45% ± 15%. Tumors were developed for 6 or 8 weeks, with varying volumes at the end of this period.

Suspensions of MNP-APS and MNP-pHLIP in PBS buffer (100 μL) were administered to mice by retro-orbital (venous sinus) route in a dose of 1 mg [Fe]/kg. Before administration, the suspensions were sonicated using Q700 Sonicator equipment with cup horn (Qsonica L.L.C, Newtown, CT, USA). The tumor-bearing mice administered 100 μL PBS were used as control. Before and 40 h after nanoparticle administration, the mice were MRI scanned.

#### 2.4.1 ^1^Н MRI

The ^1^Н MRI experiment was performed on a horizontal tomographic scanner with magnetic field intensity of 11.7 T (Bruker, Biospec 117/16 USR). The mice were anesthetized with gas anesthesia (Isofluran; Baxter Healthcare Corp., Deerfield, IL) using a Univentor 400 Anesthesia Unit (Univentor, Zejtun). The animal body temperature was maintained with a water circuit installed into the table bed of the tomographic scanner, which maintained the temperature of 30 °C on its surface. A pneumatic respiration sensor (SA Instruments, Stony Brook, NY) was placed under the lower body part, which allowed control of anesthesia depth. MR images were recorded with a receiver–transmitter ^1^H volume coil (T11440V3). High-resolution T2-weighted images of tumors in the mice (section thickness 0.5 mm; field of vision (FOV) 4.0 × 4.0 mm; matrix 512 × 512 dots) were acquired with respiratory triggering using TurboRARE T2 (Rapid Acquisition with Relaxation Enhancement) method with pulse sequence parameters: TE_eff_ = 11 ms, TR = 2500 ms, Flip Angle = 180°, RARE Factor = 42. Signal intensity (SI) was measured within the region of interest (ROI) in the tumor and standard phantom. The SI changes in T2-weighted MR images of the tumor recorded before and 40 h after MNP-APS and MNP-pHLIP administration were calculated according to the following formula (2):

(2) T2(%)=(SITpostSIPpost)(SITpreSIPpre)∗100

where T2 - is the ratio of the SI change in T2-weighted MR image of tumor before and 40 h after nanoparticle administration, %

SIT_post_- SI measured within the ROI in the tumor 40 h after nanoparticle administration;

SIP_post_ - SI measured within the ROI in standard phantom 40 h after nanoparticle administration;

SIT_pre_ - SI measured within the ROI in the tumor before nanoparticle administration;

SIP_pre_ - SI measured within the ROI in standard phantom before nanoparticle administration.

#### 2.4.2 Biological sample collection

After MRI scanning, anesthetized mice were sacrificed by cervical dislocation. Blood samples were collected in Microvette® CB 300.

The liver, spleen, kidney, lung, thymus, intact and regional lymph nodes, and tumors were dissected and weighed. The organs (50–60 mg) and tumor samples (10–50 mg) were frozen at −80 °C for iron content determination. Samples of tumors (with small, medium, and large volume extracted from randomly chosen mice in control (n = 3), MNP-APS (n = 3) and MNP-pHLIP (n = 4) groups) were taken for TEM and histological examination. For this purpose, a small piece (1 mm^3^) of tumor was immediately placed in cold 2.5% glutaraldehyde in 0.1 M Na cacodylate buffer (7.4) for TEM analysis. Another piece of the sample was placed into 10% neutral buffered formalin for histological analysis.

#### 2.4.3 Transmission electron microscopy

Fixation for TEM analysis was carried out according to Ref. [9]. Tumor samples were fixed in 2.5% glutaraldehyde in 0.1 M sodium cacodylate buffer, pH 7.4, for 2–3 h, followed by 3 washes in the same buffer for 5 min each. Then the samples were post-fixed with 1% OsO_4_ and 0.8% potassium ferrocyanide in cacodylate buffer for 1 h, rinsed 3 times in distilled water and left in 1% aqueous uranyl acetate at 4 °C for 12 h. Samples were dehydrated in ethanol series (30%, 50%, 70%, 96% and 100% for 10 min each) and acetone (twice, for 20 min), embedded in Agar 100 Resin (Agar Scientific Ltd., Essex, UK), and left to polymerize at +60°С for 2–3 days. Semi-thin sections were obtained with a Reichert–Jung ultracut ultra-microtome (Reichert-Jung, Vienna, Austria), stained with methylene blue and analyzed with an Аxioscop 40 (Zeiss, Jena, Germany) light microscope. Ultrathin sections were made using a Leica ultracut ultra-microtome (Leica, Wetzlar, Germany) and examined with a transmission electron microscope JEM-1400 (JEOL, Tokyo, Japan). For the evaluation of eosinophils inside tumor tissue, random ultrathin sections of each sample were taken and the percentage of eosinophils among random cells (n = 50 per sample) was counted (Table 3).

#### 2.4.4 ICP-AES

Tissue samples were digested in 3 mL of nitric acid and boiled until the formation of a colorless solution. The solution volume was then brought up to 10 mL by DI water and the concentration of Fe was measured by inductively coupled plasma atomic emission spectroscopy (iCAP 6300 Duo, Thermo Scientific) (Table 4). Fig. 7 shows the dependence of SI change in T2-weighed MR image of tumor after nanoparticle administration on iron concentration in the tumors determined according to ICP-AES.

#### 2.4.5 Histological analysis

For histological analysis, samples were processed on the Excelsior™ AS Tissue Processor (Thermo Scientific): samples were dehydrated in isopropanol series and embedded in HISTOMIX paraffin (Biovitrum). Tissue sections were cut into 4-5 μm-thick slices (HM 325 Rotary Microtome, Thermo Scientific). The histological slides were analyzed via optical microscope Axioskop 40 FL (Carl Zeiss) (Figs. 8 and 9). Standard hematoxylin and eosin, Perls' Prussian blue and Van Gieson's (using combined solutions of picric acid and acid fuchsin) staining protocols were used for standard analysis, iron aggregates and collagen fiber visualization.

#### 2.4.6 Immunohistochemical analysis of tumor

Formalin-fixed, paraffin-embedded tissue sections were deparaffinized by washing in two changes of xylene, then dehydrated in 100%, 95% and 70% ethanol followed by water. EDTA (pH 8.8) antigen retrieval buffer was used to unmask the antigenic epitope. Immunohistochemical staining of tumor sections was performed on BOND-MAX Automated IHC/ISH Stainer (Leica Biosystems) using anti-CD31 primary antibody (1:20, ab28364, Abcam) and EnVision FLEX/HRP (Dako). The slides were developed with EnVision FLEX DAB + CHROMOGEN (EnVision FLEX Substrate Buffer, Dako).

#### 2.4.7 Serum analysis

Mouse serum was analyzed on ARCHITECT c4000 (Abbott Diagnostics, Lake Forest, IL) using commercial kits (DiaS) (Table 5).

### 2.5 Statistical analysis

Statistical analyses were performed using a statistics program (GraphPad Prism; GraphPad Software). Data are presented as mean (SD). The data were tested by the Shapiro–Wilk test for normality. The differences between continuous variables with normal distribution were analyzed by the Student's t-test. When three groups were compared the differences among continuous variables with normal distribution were analyzed by the ANOVA, and among continuous variables with non-normal distribution by the Kruskal-Wallis test. A p value below 0.05 was considered as significant.
